# Role of PD-L1 expression as a biomarker for GEP neuroendocrine neoplasm grading

**DOI:** 10.1038/cddis.2017.401

**Published:** 2017-08-24

**Authors:** Elisabetta Cavalcanti, Raffaele Armentano, Anna Maria Valentini, Marcello Chieppa, Maria Lucia Caruso

**Affiliations:** 1 Department of Pathology, National Institute of Gastroenterology “S. de Bellis”, Research Hospital, Castellana Grotte, Bari 70013, Italy

## Abstract

Neuroendocrine neoplasms (NENs) are rare, heterogeneous and ubiquitous tumors commonly localized in the gastrointestinal tract, lung, and pancreas. The clinical behavior of NEN is highly unpredictable; in fact, low-grade cases can unexpectedly be associated with metastases. Currently, the 2010 WHO NEN classification employs histological differentiation and the proliferation index for grading tumors but fails to provide reliable prognostic and therapeutic indications. Therefore, there is an urgent need for a better characterization of G2/G3 NENs. Similar to several other tumors, NENs possess immune-escape mechanisms, but very little has yet been done to characterize this crucial aspect. There are no available data describing PD-L1 expression in these tumors. Here we provide, for the first time, evidence of PD-L1 tissue expression in gastroenteropancreatic neuroendocrine neoplasms (GEP-NENs). PD-L1 expression was significantly associated with a high-grade WHO classification (G3) (*P*<0.001) but not with gender, primary site, or lymph node status. The PD-L1 positivity rate and signal intensity are directly correlated (*P*<0.001) with a grade increase from G1 to G3. In particular in G3 cases, we observed a dichotomy between the morphology (WD- and PD-NENs) and Ki67. Moreover, our study demonstrated a significant association with the grade and PD-L1 expression levels in immune-infiltrating cells (*P*<0.001). In particular, G3 tumors are characterized by strong PD-L1 expression in both the tumor and infiltrating immune cells (*P*<0.001), reflecting an unfavorable environment for T-cell-mediated tumor aggression. These findings suggest that NENs might acquire resistance to immune surveillance by upregulating PD-L1 and inhibiting peritumoral and intratumoral infiltrating lymphocytes. Here we demonstrate that PD-L1 is currently the best-known biomarker for G3 NENs, becoming the new gold standard for G3 NEN discrimination. Furthermore, pharmacological approaches using anti-PD-1 antibodies may become the logical choice for the treatment of G3 cases with a poor prognosis.

Neuroendocrine neoplasms (NENs) are commonly localized in the gastrointestinal tract, lung, and pancreas.^1^ These rare neoplasms are heterogeneous and ubiquitous tumors defined as epithelial, with a predominant neuroendocrine differentiation, and have a reported incidence of 2.5–5 cases/100 000 people.^2, 3^ Their characteristic slow-growing feature is associated with the absence of pathognomonic symptoms, so they are difficult to diagnose: they may remain silent for years and are discovered only when they are already metastatic.^4^ The clinical behavior of NENs is highly unpredictable; in fact, unlike most tumors, low-grade cases can be associated with metastases. In 2000 and 2010, the WHO provided two different classifications of NENs based on the histopathological pattern, tumor grade, and stage intended to improve the therapeutic and prognostic evaluation.^5, 6^ Most gastrointestinal NENs are well differentiated (WD) and characterized by a slow growth rate (hence the old term carcinoid). Gastrointestinal neuroendocrine carcinomas are highly aggressive malignancies and are mainly treated with a platinum-based chemotherapy.^7^ However, at certain locations, such as the esophagus or colon, poorly differentiated NENs are more frequent than their WD counterparts.^8^ The tumor morphology and Ki67 immunostaining have been defined as prognostic factors that can subdivide gastrointestinal NENs into two or even three subgroups with significantly different survival rates.^9^ However, the two current prognostic evaluation systems fail to guide the most efficient therapeutic strategy on the basis of the histological differentiation of the tumoral cell and their proliferative index. In particular, the 2010 WHO NEN classification identified three groups: NENs G1, NENs G2, and G3, also defined as neuroendocrine carcinoma (NEC).^10, 11^ In general, the WD NENs (G1/G2) are much more common (70%) than the poorly differentiated NENs.^12^ However, some WD cases exhibit a high level of proliferation and, on the contrary, some poorly differentiated cases show a low mitotic activity. Besides, the defined range (G1: <2 mitoses/10 HPF; G2: 2–20 mitoses/10 HPF; G3: >20 mitoses/10 HPF) appears very broad, including many heterogeneous clinical, morphological and prognostic conditions.^13^ A few recent papers^14^ indicated that despite the assessment of the proliferation fraction, some tumors, identified as NECs, do not show the expected poorly differentiated morphology. These reports indicate that these patients do not respond to cisplatin-based chemotherapy, the gold standard therapeutic approach to high-grade PD-NECs,^14^ and have a better prognosis. In contrast, WD-NENs, despite mostly featuring a low/intermediate differentiation grade, may contain regions with an increased proliferation rate that places them, in accordance with the WHO, in the WHO G3 category.^15^ Specifically, G3 NENs with a WD morphology are commonly Ki67 positive (>20% but usually <55%) and respond poorly to cisplatin-based treatment. In contrast, patients with a poorly differentiated NEC, but Ki67 expression of >60%, respond to cisplatin-based chemotherapy.^16, 17, 18^ In addition, PD-NEC may also include a combined component of a conventional carcinoma (MANEC),^19^ such as a squamous cell carcinoma or adenocarcinoma, but they do not typically contain a lower-grade WD-NEN.^20, 21^ Therefore, the availability of biomarkers that could better characterize G3 GEP NENs could have crucial therapeutic implications, indicating the most efficient pharmacological strategy. Immunotherapy, which allows immune cells to attack cancer cells, results in long-term survival in patients with several different tumors, such as melanoma, non-small cell lung cancer, and renal cell carcinoma.^22^ Cancer immunotherapy has undergone major breakthroughs during the past decades, impressive results being reported by several studies.^23, 24^ One key target of cancer immunotherapies has been the PD-1–PD-L1 pathway.^25, 26^ Programmed death ligand 1 (PD-L1), also known as cluster of differentiation 274 (CD274), is expressed on many cancer cells. PD-L1 binding to PD-1 results in a pattern of inhibitory signals, which inhibits CD8^+^ T cells’ proliferation, thus blocking the antitumor immune response and hence favoring tumor growth and metastasis.^27^ PD-1 has been detected in tumor-infiltrating lymphocytes (TILs) present in the tumor microenvironment, where PD-L1 aberrant expression is associated with a poor prognosis in several human tumors.^28, 29^ Taube *et al.*^30^ demonstrated that the expression of PD-L1 in tumor cells and tumor-infiltrating immune cells was highly correlated with PD-1 expression in infiltrating lymphocytes. They also demonstrated that PD-L1-positive tumors are those that respond better to Nivolumab administration. Nivolumab is a monoclonal anti-PD-1 antibody that can block the aforementioned inhibitory signals that prevent activated T cells from attacking the cancer. Anti-PD-1 administration may improve the prognosis of patients characterized by both tumor cells’ PD-L1 expression and TILs, as shown by multiple studies.^31, 32^ PD-L1 expression on tumor cells and immune-infiltrating cells, such as tumor-infiltrating macrophages, appears to be a crucial predictor of the antitumor T cells’ response. Importantly, in most clinical trials, the response rates of various tumor types to PD-L1/PD1 targeting therapies were correlated with the immune-histochemical expression of PD-L1, suggesting that PD-L1 expression could be a predictive biomarker of the tumor sensitivity to immunotherapy.^33^ Immunological approaches against NENs have not yet been explored, but in the present manuscript, we provide data that may better support the possible benefit deriving from the revolution in immunotherapy as regards the clinical evaluation of NENs and the identification of the most effective pharmacological strategy. PD-L1 tissue expression has not been addressed in gastroenteropancreatic NENs (GEP-NENs). Here we provide evidence of PD-L1 tissue expression in different NENs. PD-L1 expression, together with Ki67, offers a new and efficient benchmark for a broad range of grading of GEP-NENs. Therefore, results of the present study indicate that PD-L1 tissue expression may be the best GEP/NEN morphological characterization and grading marker. Furthermore, Nivolumab administration may become the pharmacological choice for Ki67>60% PD-NENs, identified in this study as the tumors with the highest PD-L1 expression.

## Results

### Patient population

The patients characteristics of the 57 cases of NETs are summarized in Table 1. Patients median age was 56.5 years (range: 30–87): 25 were females (44%) and 32 were males (56%) (male-to-female ratio 1.47). Primary sites included the small intestine (28%), stomach (17.5%), liver (17.5%), gall bladder (2%), colon (12%) pancreas (17.5%), ampulla of vater (2%), and skin (3.5%). As some cases were graded before the 2010 WHO classification, we performed a Ki67 proliferation assay for all cases examined. The results obtained confirmed the previous classification, with only one case switched from G1 to G2: a pancreatic NEN, previously assessed as G1 based on morphological evaluation alone was graded as G2 on the 8% Ki67 and mitotic index <2%. The 57 cases of NENs included in the present study are classified as follows: 39 grade 1 (68%), 9 grade 2 (16%), and 9 grade 3 (16%). Only one G1 case was first diagnosed as derived from liver cancer while a further evaluation revealed that the primary tumor site was the small intestine (ileum). Two cases, at the duodenal bulb (G1) and colon (G3), were associated with epithelial cancer: one was associated with gastric carcinoma and the other to a poorly differentiated adenocarcinoma of the left colon. Lymph node metastases and visceral peritoneum invasion were detected only in two NEN G1 (head of the pancreas and ileum).

### Correlation between PD-L1 expression and grading

**Figure 1:**
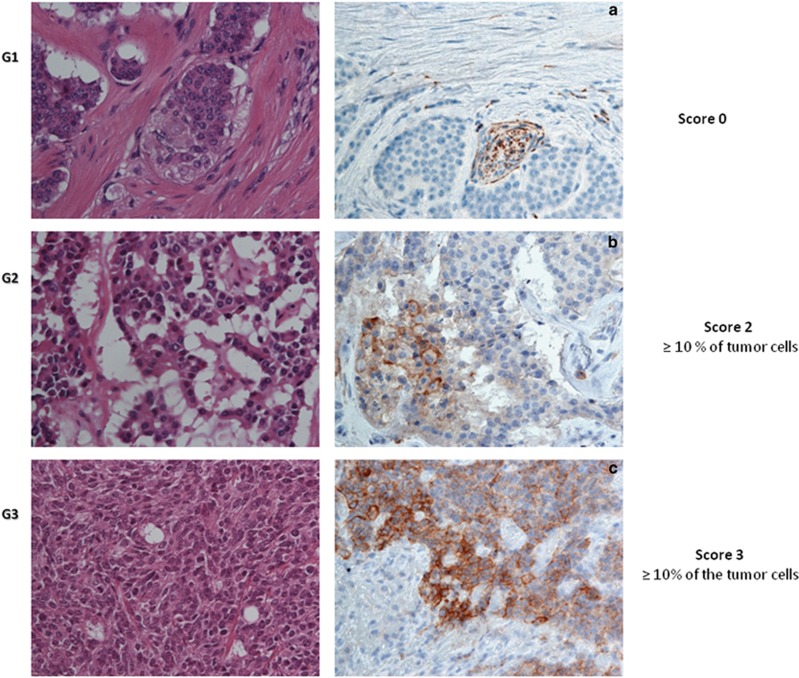
Representative patterns of PD-L1 staining intensity and grade are shown: (**a**) G1, IHC negative; (**b**) G2, medium expression; (**c**) G3, strong expression (magnification × 40)

**Figure 2:**
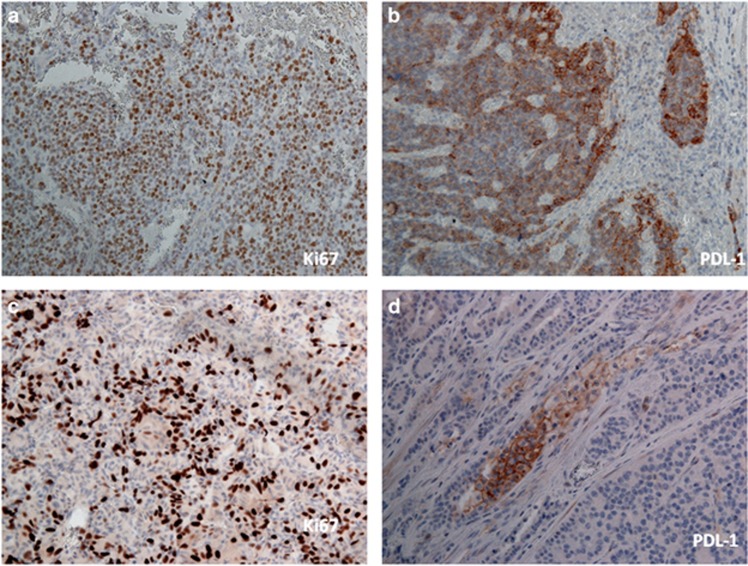
Correlation between the PD-L1 tissue expression and Ki67 index in G3 NENs: (**a** and **b**) representative G3 cases with a 3+ PD-L1 expression signal and Ki67 of >60% (**c** and **d**) G3 cases with a 2+ score and Ki67 of 45%

**Figure 3:**
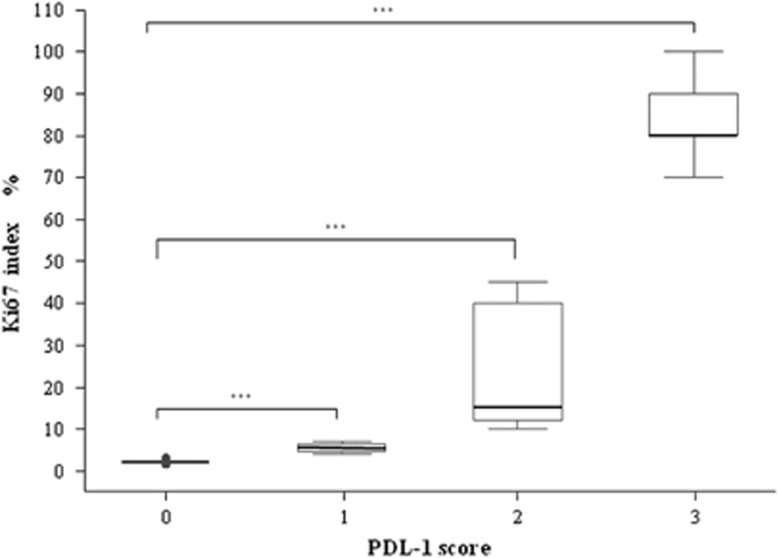
Correlation between the PD-L1 score and Ki67 index in NEN patients (rho=0.959, *P*<0.001): Box plots of PD-L1 score and Ki67 index (expression %) ***P<0.001

We evaluated the cell membrane PD-L1 expression in tissues from 57 patients with NENs. Among these, 41 cases resulted negative (72%) while 16 cases were positive (28%). The relationship between PD-L1 expression and gender was not significant (*P*=0.345; Table 2). Importantly, PD-L1 expression was absent in all cases with WD-NENs (G1), while PD-L1 positivity in tumor cell membranes was detected in 7 G2 cases (78%) and 9 G3 cases (100%). Therefore, PD-L1 expression is significantly associated with a high-grade WHO classification (G3) (*P*<0.001; Table 2) but not with gender, primary site or lymph nodes metastatic status. The PD-L1 staining intensity score on neoplastic cells was different among the three grades (Figure 1). In particular, G2 patients showed a weak signal in 4 cases (44%), medium signal in 3 cases (34%) and no signal in 2 cases (22%). In G3 patients, the PD-L1 signal was medium in 2 cases (22%) and strong in 7 cases (78%) (Table 3). There was no G3 patient without a PD-L1 signal. These data indicate that the PD-L1 positivity rate and signal intensity are directly correlated with the grade increase (*P*<0.001) from G1 to G3 (Figure 1). In addition, we assessed the possible correlation between PD-L1 and the Ki67 index. In particular in G3 cases (all PD-L1 positive), we observed a dichotomy between the morphology (WD- and PD-NENs) and Ki67. Based on the PD-L1 signal intensity, we created a score (Table 3) from 0 to 3+. As stated above, all G3 cases were PD-L1 positive and 2 cases had a score of 2+ (33%) while the rest (7 patients) had a score 3+ (67%). Patients with score 2+ for the PD-L1 expression signal had a Ki67 in the 20–60% range, while patients with score 3+ had a Ki67 >60% (Figure 2). PD-L1 tissue expression was significantly correlated with the Ki67 index (rho=0.959), and we found a significant positive trend of association with the Ki67 proliferation index (*P*<0.001). Furthermore, we highlighted significant differences between the PD-L1 score and the Ki67 index and, in particular, observed a remarkable difference between individual Ki67 mean levels and the PD-L1 score (*P*<0.001; Figure 3).

### PD-L1 expression in peritumoral and intratumoral immune-infiltrating cells

**Figure 4:**
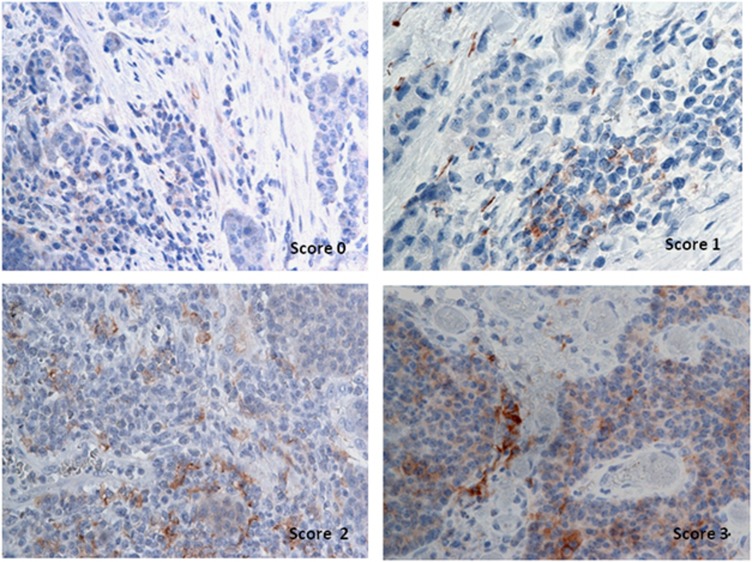
Representative PD-L1 staining intensity in peritumoral and intratumoral immune-infiltrating cells: (**a**) no staining (0), (**b**) weakly positive staining (1+), (**c**) moderately positive staining (2+), (**d**) strongly positive staining (3+)

PD-L1 expression was also detected in peritumoral and intratumoral immune-infiltrating cells. Immune-infiltrating cells were most frequently located at the interface between tumor cells and stroma, with similar morphological features to the lymphoepithelial lesions frequently present in MALT lymphomas. In our study, we highlighted a variable PD-L1 expression by grade in immune-infiltrating cells, as shown in Figure 4 and Table 2. In particular, 74.4% of G1 patients did not express PD-L1-positive infiltrating cells, suggesting that G1 patients may have different tumor immune interference mechanisms: the 25.6% of positive G1 patients showed a weak PD-L1 signal in 5 (50%, 13% of the total) and moderate in the other 5 (50%, 13% of the total) (Table 3). In G2 patients, PD-L1 expression was detected in 77.8% of the infiltrating cells: 4 patients were PD-L1 (45%) negative, 2 showed a weak (22%), and 3 a moderate expression (33%). In contrast, infiltrating cells were all positive (100%) to PD-L1 in G3 patients (Table 2), 78% with intense and 22% with moderate expression (Table 3). Our data demonstrate that G3 patients are characterized by a strong PD-L1 expression in both tumor and infiltrating immune cells (*P*<0.001; Table 2), reflecting an unfavorable environment for T-cell-mediated tumor aggression.

## Discussion

The expression of PD-L1 is significantly associated with the histopathological grade and poor clinical outcomes in various malignant tumors, such as gastric cancer, hepatocellular carcinoma, renal cell carcinoma, esophageal cancer, pancreatic cancer, ovarian cancer, and bladder cancer.^34, 35, 36, 37^ The PD-L1-positive rate was ∼30% in melanoma cases and 25–36% in non-small cell lung cancer; in NENs, we found 28% cases.^38, 39^ Some studies explored, by immunohistochemistry (IHC), PD-L1 expression in different tumors and demonstrated that PD-L1 membranous expression on tumor cells and/or infiltrating immune cells is correlated to a better chance of response to anti PD-1 drugs.^40, 41^ However, the prognostic role of PD-L1 remains controversial, mainly due to tumor heterogeneity, variability in the assays, location of intratumoral expression, and cutoff values for positive *versus* negative expression. Therefore, in the present study we investigated the impact of PD-L1 tissue expression on the grading of 57 GEP/NEN patients, associating PD-L1 expression with the morphological characterization. The characteristics of indolent or aggressive GEP-NEN were determined by tumor grade, differentiation, and proliferation status.^42^ The current WHO 2010 grading system was designed to provide clinicians with an effective tool for patients stratification and clinical management. Years later, this WHO classification fails to provide an efficient cutoff between G1, G2, and G3 likely because existing biomarkers discriminate only partially between different grades. Currently, the Ki67 expression level is associated with different prognoses and can only partially indicate the most efficient pharmacological strategy. Similarly, high-grade G3 NECs are defined by morphology and Ki67 expression (>20% but usually <50%).^15^ The broad interval indicated by Ki67 expression for G3 disease (21–100%) may include a variety of different neoplasms, with potentially different responses to therapies.^43^ Overall, the morphology-based classification of different categories is still challenging, especially for G3 cases. Our results indicate, for the first time, that PD-L1 tissue expression is present in 100% of G3 NENs, those cases in which the Ki67 expression is an unreliable benchmark for treatment choice and intratumoral grade heterogeneity assessment. The terminology of ‘neuroendocrine carcinoma’ for G3 neoplasms implies that they are histologically poorly differentiated, but some morphologically WD-NETs also have proliferation rates (usually the Ki67 index) that meet the threshold for G3 NECs. A recent landmark study showed that G3 NECs with a Ki67 index of <55% do not respond to cisplatinum-based chemotherapy, unlike G3 NECs with a Ki67 index >55%.^44, 45^ This supports the concept that the current WHO G3 category is heterogeneous and that tumors at the lower end of the G3 range are, in fact, WD-NETs with an elevated proliferation rate (or ‘high-grade, WD neuroendocrine tumors’). Membranous PD-L1 expression by tumor cells and immune infiltrates varied significantly by grade and was significantly associated with the high-grade WHO classification (G3) (*P*=0.001) but not with gender, primary site, or number of metastatic sites. Our findings highlight that the expression of PD-L1 is present in the proliferative, aggressive G3 type of GEP-NET and, only in G3, shows a moderate-to-strong signal. In particular, poorly differentiated NENs (NEN G3 and NEC) have a constant positive PD-L1 membrane expression that increases with the tumor aggressiveness. Consistently with previous studies based on different tumors,^46, 47^ PD-L1 expression is correlated with a more aggressive subset of NENs identified using morphological data and routine staining. In accordance with this possibility, PD-L1 expression has been reported as the most important predictor of the responsiveness of various cancers to PD-L1 or PD-1 blocking antibodies.^35, 48^ Moreover, our study demonstrated a significant association with grading and PD-L1 expression levels in infiltrating immune cells (*P*=0.001). PD-L1 expression on tumor cells and tumor-associated immune cells was also positively correlated with the progression of human malignant tumors.^49^ PD-1 is expressed by activated T cells^50^ and its engagement with PD-L1 inhibits TCR-mediated activation of IL-2 production and T-cell proliferation. These findings suggest that NENs might acquire resistance to immune surveillance by upregulating PD-L1 and inhibiting peritumoral and intratumoral infiltrating lymphocytes. Thus this hypothesis provides a strong rationale for PD-1/PD-L-targeted immunotherapy for NENs. Recent studies^51, 52^ have demonstrated that PD-L1 has a critical role in regulatory T-cell (Treg) development and functional maintenance. Increases in FOXP3+Treg infiltration and PD-L1 expression have been revealed in gastric cancer tissues, colorectal carcinoma, and breast cancer. High PD-L1 expression and increased FOXP3+Treg infiltrates were both associated with high histological grade, and tumors with concomitant high expression levels of the two markers had the worst prognosis. Recently, it has been reported that PDL-1 blockade improves antitumor immunity and offers a promising cancer immunotherapy approach. The blockade of PD-L1 in non-small cell lung cancer might be one strategy to pursue for future immunotherapy.^53^ Several clinical studies have suggested that TILs have a critical role and a prognostic significance in certain human tumors. Recently, PD-L1 has also been found to be expressed in a broad range of cancers. The observation in our study that PDL-1 is overexpressed in NENs (G2–G3) indicates that tumor-related PD-L1 may be linked to a malignant potential and contribute to tumor progression by providing a protective mechanism against immune surveillance. The significant association between PD-L1 expression and grading confirms that tumor-related PD-L1 expression is indeed relevant to tumor development. In our study, we observed high levels of PD-L1 expression on the infiltrating immune cells of PD-NENs. Thus it is possible that the tumoral milieu promotes macrophages PD-L1 expression that, in turn, inhibits the function of TILs, favoring tumor progression. We recognize that 57 NENs cases, although rare tumors, are a small cohort, but the incidence of PD-L1-positive patients with grade G3 warrants a larger future study. PD-L1 expression could become the new gold standard for G3 NENs discrimination. To the best of our knowledge, this is the first report evaluating the characteristics and grading of PD-L1 tissue expression in patients with GEP-NENs, paving the way for a new and more efficient WHO classification. In fact, it is expected that a new WHO classification, to be published in 2017, should provide a new NEC category G4 (Ki67>55%), as recently proposed by Fazio *et al.*^54^ This subdivision, correlated with a timely diagnosis, may ensure the implementation of appropriate treatment and have a substantial impact on prognosis. Furthermore, based on these observations, pharmacological approaches using anti-PD-1 antibodies may become the logical choice for the treatment of G3 cases, currently burdened by a poor prognosis. Immunotherapy might be a valid alternative or support to other therapies in NENs. Moreover, PD-L1 might be a useful prognostic biomarker in GEP-NENs. At the moment, it seems that high-grade tumors might be the targets. In this context, it is important to carefully delineate those tumors that might better respond to this type of treatment.

## Materials and methods

### Patients’ information

In total, 57 cases of NENs who underwent curative surgery^32^ or endoscopic resection^25^ from January 2006 to December 2016 were reviewed at the National Institute of Gastroenterology Research Hospital ‘Saverio de Bellis’. The following clinicopathological characteristics were collected for all patients: age, gender, primary site, tumor grade (according to the 2010 WHO classification), and metastasis (Table 1).

### Pathological assessment

The pathological diagnosis was confirmed by two pathologists who reviewed FFPE tissue sections stained with hematoxylin and eosin (H&E), and a representative paraffin block from each specimen was chosen for IHC analysis. Tumor size was measured either grossly (obtained from pathology reports) or microscopically; no case measured >5 cm. The criteria for further classification and grading were based on both the morphological features of the tumor and the proliferation rate. On H&E and PAS mucin-stained sections, the cytological characteristics of cells, growth patterns, presence of ulcerations, level of infiltration in the organ, and infiltration of mucosa were evaluated. In addition, we evaluated perineural infiltration, vascular permeation, presence of necrosis, intratumoral and peritumoral lymphocyte infiltration, and lymph node metastases (Table 1). Intratumoral necrosis, on the contrary, was present only in NEC ulcerated areas. Regardless of the size of the tumor, the WD tumors showed, similar to NEN G3, a clear infiltration pattern and sometimes frequent vascular permeation images and perineural infiltration. All the cases were reviewed to confirm the diagnoses according to the WHO 2010 on sections stained with H&E and on the basis of the Ki67 percentage before starting the investigation. Therefore, as we had several cases dating back to 2010 WHO classification, we have reviewed all cases and performed again a Ki67 proliferation assay for all cases examined. The results obtained confirmed by two pathologists classify all cases.

### Immunohistochemistry

IHC analysis for PD-L1 was performed in the FFPE of 57 patients with NENs. Tumor sections of 4 mm were freshly cut and dried at 60 °C for 30 min. IHC analysis was carried out in sections after deparaffinization for 30 min and then rehydration in grades of alcohol. Antigen retrieval was performed at 90 °C for 20 min with Tris–borate–EDTA Buffer. To assess the PD-L1 staining employed for the present study, two different antibodies (clone E1L3N, Cell Signaling Technology, Beverly, MA, USA, at 1:600 dilution and clone SP142, Roche/Ventana Medical Systems, Tucson, Arizona, USA at 1:100 dilution) were evaluated on the NENs, using an automated autostainer (cat. K5007, Dako, Glostrup, Denmark). The Real Envision DAB Substrate Kit (DAKO) was used according to the manufacturer’s instructions. Human placenta was included as positive control. The staining pattern of both antibodies was as expected: cellular membranous/submembranous and cytoplasmic with occasional dots, corresponding to the PD-1/PD-L1 interaction sites. The E1L3N antibody clone was preferred for further evaluations primarily because of the better signal-to-noise results. Ki67 IHC was carried out again on all cases on paraffin-embedded sections using an Ab anti-Ki67 diluted 1:100 (Mib-1; DAKO) following the manufacturer’s instructions. All cases were revised and re-evaluated and then assigned a precise proliferation index number that encompassed the amplitude limit of the WHO range. All IHC stained sections were initially evaluated and scored by two pathologists; discrepancies in the interpretation of scoring were resolved by consensus. PD-L1 expression was evaluated both on tumor cells and inflammatory cells as intratumoral and peritumoral in location. PD-L1 expression can be observed as a cytoplasmic or membranous immunoreactivity. The proportion of PD-L1-positive cells was estimated as the percentage of total tumor cells; tumor cells typically showed membranous staining with a variable cytoplasmic staining component. In literature, tumors with ⩾1% of tumor cells stained either in membrane or cytoplasm were considered positive for PD-L1 regardless of the intensity of staining.^55^ In the present study, all PD-L1 staining results were scored as both the approximate percentage of positive tumor cells only in the membranous site and the intensity of overall staining. In particular, the expression of PD-L1 was scored: 0: (no staining) negative; 1: weak expression, but weaker than the placenta membrane, staining in <10% of tumor cells; 2: moderate expression in ⩾10% of tumor cells; and 3: strong more than placenta membrane staining in ⩾10% of the tumor cells. For the data assessment, our cases were considered to be positive for PD-L1 expression only if they had scores of 2+ or 3+ (Figure 1). Moreover, the intensity of PD-L1 staining in intratumoral and peritumoral immune-infiltrating cells was judged on a semiquantitative scale of 0–3+: no staining (0), weakly positive staining (1+), moderately positive staining (2+), and strongly positive staining (3+).

### Statistical analyses

Correlations among the PD-L1 expression score of tumor cells, immune-infiltrating cells, and grading were statistically analyzed by *t*-test or Fisher’s exact test and *χ*^2^ test. All *P*-values were determined by two-sided tests and *P*-values<0.05 were considered significant. Statistical analyses were performed with StataCorp. 2007 Stata Statistical Software: release 10 (StataCorp LP, College Station, TX, USA).

## Figures and Tables

**Table 1:** Baseline patients’ characteristics

**Patients characteristics**	***N***	**%**
*Gender*		
Male	34	60
Female	23	40
		
Age, years (median, range)	56.5	
		
*Localization*		
Stomach	10	17.5
Liver	10	17.5
Gall bladder	1	2
Colon	7	12
Pancreas	10	17.5
Small intestine	16	28
Ampulla of vater	1	2
Skin	2	3.5
		
Grade WHO classification		
G1	39	68
G2	9	16
G3	9	16
		
Lymph node metastasis, yes	2	3.5
Perineural infiltration, yes	47	81
Vascular permeation, yes	3	5.2
Necrosis, yes	9	15.5

**Table 2:** PD-L1 expression on different grade of NEN and infiltrating immune cells

	**PD-L1 expression**			
	**Positive,** ***n*** **(%)**	**Negative,** ***n*** **(%)**	**Total,** ***n*** **(%)**	***P*****-value**
*Gender*				
Male	9 (28)	23 (72)	32	0.345
Female	10 (40)	15 (60)	25	
				
*Histological grade (WHO 2010)*				
Grade 1	0	39 (100)	39	0.0001
Grade 2	7 (78)	2 (22)	9	
Grade 3	9 (100)	0	9	
				
*Infiltrating immune cells intratumoral/peritumoral*				
Grade 1	10 (25.6)	29 (74.4)	39	0.0001
Grade 2	7 (77.8)	2 (22.2)	9	
Grade 3	9 (100)	0	9	

**Table 3:** PD-L1 intensity score on neoplastic cells and intratumoral and peritumoral immune-infiltrating cells

	**Staining**				
	**Negative**		**Positive**		
**PD-L1 intensity score neoplastic cells**	**0 (absent)**	**1 (weak)**	**2 (medium)**	**3 (strong)**	***P*****-value**
	***n*****(%)**	***n*****(%)**	***n*****(%)**		**0.0001**
Grade 1	39 (100)	0	0	0	
Grade 2	2 (22)	4 (44)	3 (34)	0	
Grade 3	0	0	2 (22)	7 (78)	
